# Exploring ammonium tolerance in a large panel of *Arabidopsis thaliana* natural accessions

**DOI:** 10.1093/jxb/eru342

**Published:** 2014-09-09

**Authors:** Asier Sarasketa, María Begoña González-Moro, Carmen González-Murua, Daniel Marino

**Affiliations:** ^1^Department of Plant Biology and Ecology, University of the Basque Country (UPV/EHU), Apdo. 644, E-48080 Bilbao, Spain; ^2^Ikerbasque, Basque Foundation for Science, E-48011 Bilbao, Spain

**Keywords:** Ammonium, *Arabidopsis thaliana*, glutamate dehydrogenase, glutamine synthetase, natural variation, nitrate, nitrogen.

## Abstract

Plants are dependent on exogenous nitrogen (N) supply. Ammonium (NH_4_^+^), together with nitrate (NO_3_^–^), is one of the main nitrogenous compounds available in the soil. Paradoxically, although NH_4_^+^ assimilation requires less energy than that of NO_3_^–^, many plants display toxicity symptoms when grown with NH_4_^+^ as the sole N source. However, in addition to species-specific ammonium toxicity, intraspecific variability has also been shown. Thus, the aim of this work was to study the intraspecific ammonium tolerance in a large panel of *Arabidopsis thaliana* natural accessions. Plants were grown with either 1mM NO_3_^–^ or NH_4_^+^ as the N source, and several parameters related to ammonium tolerance and assimilation were determined. Overall, high variability was observed in *A. thaliana* shoot growth under both forms of N nutrition. From the parameters determined, tissue ammonium content was the one with the highest impact on shoot biomass, and interestingly this was also the case when N was supplied as NO_3_^–^. Enzymes of nitrogen assimilation did not have an impact on *A. thaliana* biomass variation, but the N source affected their activity. Glutamate dehydrogenase (GDH) aminating activity was, in general, higher in NH_4_^+^-fed plants. In contrast, GDH deaminating activity was higher in NO_3_^–^-fed plants, suggesting a differential role for this enzyme as a function of the N form supplied. Overall, NH_4_^+^ accumulation seems to be an important player in *Arabidopsis* natural variability in ammonium tolerance rather than the cell NH_4_^+^ assimilation capacity.

## Summary

Ammonium nutrition is toxic to many plants. *Arabidopsis* displays high intraspecific variability in ammonium tolerance (shoot biomass), and ammonium accumulation seems to be an important player in this variability.

## Introduction

Plants have a fundamental dependence on inorganic nitrogen (N), and intensive agriculture requires the use of N compounds to supplement the natural supply from the soil. Indeed, >100 Mt of nitrogenous fertilizers are added to the soil worldwide annually (Good and Beatty, 2011). In part because of the intense use of fertilizers, agriculture is now a dominant force behind many environmental threats, including climate change and degradation of land and fresh water (Foley *et al.*, 2011; Tilman *et al.*, 2011). Moreover, recent studies suggest that agricultural output would need to roughly double to meet the expected demand associated with the increase in the world’s population (FAO, 2009).

Nitrate (NO_3_^−^) and ammonium (NH_4_^+^) are the main forms of N available for plants. There is a serious concern regarding NO_3_^−^ loss in the field, giving rise to soil and water pollution. Moreover, incomplete capture and poor conversion of nitrogen fertilizer also causes global warming through emissions of nitrous oxide. Due to these detrimental effects of adding high NO_3_^−^ concentrations to ecosystems (Gruber and Galloway, 2008), the potential of NH_4_^+^ as an N source for agriculture has been reconsidered alongside the search to improve N use efficiency (NUE) while mitigating the impact of agriculture (IPCC, 2007). Similarly, lowering fertilizer input and breeding plants with better NUE without affecting yield is a main goal for research in plant nutrition (Xu *et al.*, 2012).

Plants have differential N source preference, but this depends not only on their genetic background but also on a wide and dynamic range of environmental variables including soil pH, temperature, etc. Thus, a robust classification of plants species adapted to NO_3_^−^ or NH_4_^+^ does not exist. However, it appears that most non-bred plants preferentially take up NH_4_^+^ (Bloom *et al.*, 1993; Kronzucker *et al.*, 2001). Moreover, crop species have traditionally been bred under nitric or combined N nutrition, provoking a negative selection pressure towards NH_4_^+^ assimilation, and this undoubtedly is one of the reasons they prefer NO_3_^−^, although NO_3_^−^ must be taken up against an electrochemical gradient and then be reduced to NH_4_^+^ with the consequent energy cost (Kronzucker *et al.*, 2001). In this sense, NH_4_^+^ nutrition has been generally considered as toxic for plants, particularly when NH_4_^+^ is supplied as the sole N source. Indeed, NH_4_^+^ is also toxic to animals and fungi when present in excess amounts (Britto and Kronzucker, 2002).

Ammonium toxicity syndrome in plants includes several symptoms, among others leaf chlorosis, ion imbalance, hormone deregulation, disorder in pH regulation, decrease in net photosynthesis, and changes in metabolite levels including amino acids, organic acids, and carbohydrates. At the whole-plant level, a reduction in plant growth with increasing external NH_4_^+^ concentrations, as compared with NO_3_^−^ nutrition, is a common effect of NH_4_^+^ nutrition (Cruz *et al.*, 2006). Biomass reduction has been associated with carbohydrate limitation for growth due to excessive sugar consumption for NH_4_^+^ assimilation and to the energy costs of futile transmembrane NH_3_/NH_4_^+^ cycling in root cells (Coskun *et al.*, 2013). Indeed, plant growth is probably the best indicator of NH_4_^+^ stress as it is a comprehensive measure of the physiology of the plant as a whole (Cruz *et al.*, 2006; Dominguez-Valdivia *et al.*, 2008; Ariz *et al.*, 2011).

Substantial variations in NH_4_^+^ tolerance have been observed amongst closely related species (Monselise and Kost, 1993) and even within species (Rauh *et al.*, 2002; Cruz *et al.*, 2011; Li *et al.*, 2011), suggesting the evolution of highly distinct mechanisms to cope with this stress. The strategies plants deploy to avoid NH_4_^+^ toxicity include enhancing NH_4_^+^ assimilation and increasing the efflux outside the cell or into the vacuole. Nevertheless, at present there is no consensus as to which traits confer NH_4_^+^ tolerance or sensitivity to plants. Ammonium assimilation mainly occurs via the glutamine synthetase/glutamate synthase cycle (GS/GOGAT). However, it seems that other alternative pathways could be involved in ammonium assimilation when NH_4_^+^ is supplied as the sole source of N. Although controversial, under these conditions, glutamate dehydrogenase (GDH), that catalyses the reversible deamination of glutamate to 2-oxoglutarate, might be collaborating in NH_4_^+^ assimilation (Skopelitis *et al.*, 2006; Setien *et al.*, 2013).

*Arabidopsis thaliana* and the *Brassicaceae* family are considered to be a species, and a family, sensitive to NH_4_^+^. Most of the works focused on NH_4_^+^ toxicity in *Arabidopsis* have compared plants fed with NO_3_^−^ versus plants fed with a combined nutrition of NO_3_^−^ supplemented with increasing concentrations of NH_4_^+^. Studies where *Arabidopsis* has been grown under long-term ammonium supply as the sole N source are scarce and have shown how NH_4_^+^ causes a retardation of seedling growth or a dramatic reduction in plant biomass (Rauh *et al.*, 2002; Hoffmann *et al.*, 2007; Helali *et al.*, 2010). Also, recent genetic approaches have been useful to identify new molecular players involved in the signalling pathways that lead to NH_4_^+^ sensitivity, for example a GDP-mannosepyrophosphorylase enzyme (Qin *et al.*, 2008) or the ammonium transporter AMT1:3 (Lima *et al.*, 2010).

Overall, the evolutionary trade-off between high productivity, adaptation to low-nutrient environments, and the use of ammonium as fertilizer is a challenge to most plant cultivars that have been selected under non-limiting NO_3_^−^ or combined NH_4_^+^/NO_3_^−^ fertilization (Presterl *et al.*, 2003; Xu *et al.*, 2012). Approaches based on natural variation have become an important means to study plant adaptation to the environment. In *Arabidopsis*, it has already been reported that a plant’s response to N availability is dependent on both the genotype and the N fertilization level (Loudet *et al.*, 2003), and natural variation has been observed for N remobilization during seed filling, among others (Masclaux-Daubresse and Chardon, 2011). Thus, the present work compares the natural intraspecific variability of *A. thaliana* grown under a low NO_3_^−^ or NH_4_^+^ supply, focusing on the importance of N assimilation mechanisms in relation to the differential N source provided.

## Materials and methods

### Experimental procedures and growth conditions

Forty-seven *A. thaliana* world natural accessions lines (http://publiclines.versailles.inra.fr/naturalAccession/index) were used throughout the study. Seeds were directly sown in 37cm^3^ pots containing autoclaved perlite:vermiculite substrate mixture (1:1, v/v).

Seeds were cold-treated during 4 d in the dark at 4 ºC and then transferred into a controlled-conditions phytotron: 14h, 200mol m^–2^ s^–1^ light intensity, 60% relative humidity (RH), and 22 ºC day conditions, and 10h, 70% RH, and 18ºC night conditions. Pots were initially misted with a modified Murashige and Skoog (MS) solution containing 0.5mM NH_4_NO_3_. Nine days after transfer into the growth chamber, a single seedling was retained per pot and treatment was initiated. Plants were irrigated three times a week with a modified MS solution (3mM CaCl_2_, 1.25mM KH_2_PO_4_, 1.5mM MgSO_4_, 5mM KCl, 0.085mM Na_2_EDTA, 0.5mM MES, 5 μM KI, 0.1 μM CuSO_4_, 100 μM MnSO_4_, 100 μM H_3_BO_3_, 0.1 μM CoCl_2_, 100 μM FeSO_4_, 30 μM ZnSO_4_, and 0.1 μM Na_2_MoO_4_) with 0.5mM Ca(NO_3_)_2_ or 0.5mM (NH_4_)_2_SO_4_ as N source. NH_4_^+^-fed plants were supplemented with 0.5mM CaSO_4_ to compensate the Ca^2+^ supplied together with the NO_3_^–^.

Thirty days after transfer into the growth chamber, rosette biomass was recorded and leaves were immediately frozen in liquid nitrogen and stored at –80 ºC.

### Determination of ammonium and total amino acids content

Aliquots of ~25mg of frozen material were ground to powder with liquid nitrogen and homogenized with 800 μl of ultrapure water. Samples were then incubated at 80 ºC during 5min and centrifuged at 4000 *g* and 4 ºC for 20min, and supernatants were recovered.

Total free amino acids were determined by the ninhydrin method (Yemm and Cocking, 1955). Ammonium content was determined by using the colorimetric method based on the phenol hypochlorite assay (Berthelot reaction).

### Protein extraction

Proteins were extracted as described in Gibon *et al.* (2004). Briefly, leaves (~40mg per sample) were homogenized using a mortar and pestle with 0.8ml of extraction buffer [10mM MgCl_2_, 1mM EDTA, 1mM EGTA, 10mM dithiothreitol (DTT), 0.1% Triton X-100, 10% glycerol, 0.05% bovine serum albumin (BSA), 0.5% polyvinylpolypyrrolidone (PVPP), 50mM HEPES pH 7.5] in the presence of a cocktail of proteases inhibitors [1mM phenylmethylsulfonyl fluoride (PMSF), 1mM ε-aminocaproic acid, 10 μM leupeptin, 1mM benzamidine]. Samples were then centrifuged at 4000 *g* for 30min at 4 ºC and the supernatants recovered. Protein content of the supernatants was quantified by the Bradford assay (Bradford, 1976).

### Enzyme activities

The GS reaction was measured at 30 ºC in a reaction buffer containing: 50mM TRIS-HCl (pH 7,6), 20mM MgSO_4_, 8mM sodium glutamate, 6mM hydroxylamine, 4mM Na_2_-EDTA, and 8mM ATP. The reaction was stopped by adding 0.12M FeCl_3_, 0.5M trichloroacetic acid (TCA), and 2 N HCl. Samples were centrifuged at 13 200 *g* for 5min, and the absorbance of γ-glutamyl monohydroxamate (γ-GHM) was measured at 540nm.

GDH activity was determined in the aminating direction in a reaction buffer containing 100mM TRIS-HCl (pH 8), 1mM CaCl_2_, 13mM 2-oxoglutarate, 50mM (NH_4_)_2_SO_4_, and 0.25mM NADH, and in the deaminating direction in 100mM TRIS-HCl (pH 9), 1mM CaCl_2_, 30mM glutamic acid, and 0.25mM NAD. Both kinetic activities were monitored spectrophotometrically at 30 ºC by consumption of NADH (amination) or appearance of NADH (deamination) at 340nm.

Nitrate reductase (NR) activity was measured at 30 ºC. The reaction medium consisted of 50mM HEPES-KOH, pH 7.6, 5mM KNO_3_, 0.2mM NADH, 10 μM FAD phosphate, 1mM DTT, 20mM EDTA. The reaction was started by adding 50 μl of protein extract to 250 μl of reaction medium and stopped by adding 32 μl of 50mM zinc acetate. Then, samples were centrifuged, 100 μl of supernatant was recovered, 8 μl of 50mM phenacin metosulphate added, and the samples incubated for 20min at room temperature. Finally, 80 μl of 1% sulphanilamide in 3M HCl and 80 μl of 0.02% *N*-(1-naphthyl)ethylenediamine dihydrochloride were added and the absorbance determined at 546nm.

### Western blotting

Sodium dodecyl sulphate–polyacrylamide gel electrophoresis (SDS–PAGE) was performed in a 1.5mm thick 10% (w/v) resolving gel and a 4.6% acrylamide (w/v) stacking gel in a vertical electrophoresis cell (Mini- Protean III; Bio-Rad) at 150V for 150min. Gels were electroblotted onto nitrocellulose membrane for 75min at 100V in a Mini Trans-Blot Electrophoretic Transfer Cell (Bio-Rad). Blots were blocked in 5% (w/v) skim milk in 20mM TRIS-buffered saline at 4 °C for 1h. α-GDH (1:5000), α-GS (1:2000), and α-NR (1:1000; Agrisera, Sweeden) were used as primary antibodies. The secondary antibody was goat anti-rabbit horseradish peroxidase conjugate (1:50 000, Sigma-Aldrich, St. Louis, MO, USA). Immunoreactive bands were visualized with a highly sensitive chemiluminescent substrate for peroxidase detection (GE Healthcare Europe GmbH, Freiburg, Germany).

### Data analysis

Data analyses were carried out using SPSS 17.0 (Chicago, IL, USA). Statistical differences between nitrate and ammonium nutrition for each accession and variable were assessed comparing the mean values by paired *t*-test. To test the connectivity between variables, Pearson’s correlation coefficient was calculated for *P*≤0.05. Multiple regressions provided a view of the relationship between a trait and shoot biomass independent of other correlated traits. Multiple regression estimations can suffer from multicollinearity wherein highly correlated traits might act redundantly. Thus, to help in interpretation, Akaike’s information criterion (AIC) was also used to determine the ‘best’ model by rewarding added explanatory power but penalizing the inclusion of additional terms. This provides the simplest model with the least collinearity and, thus, supposedly, the best estimation of selection (Shaw and Geyer, 2010).

## Results

**Fig. 1.**
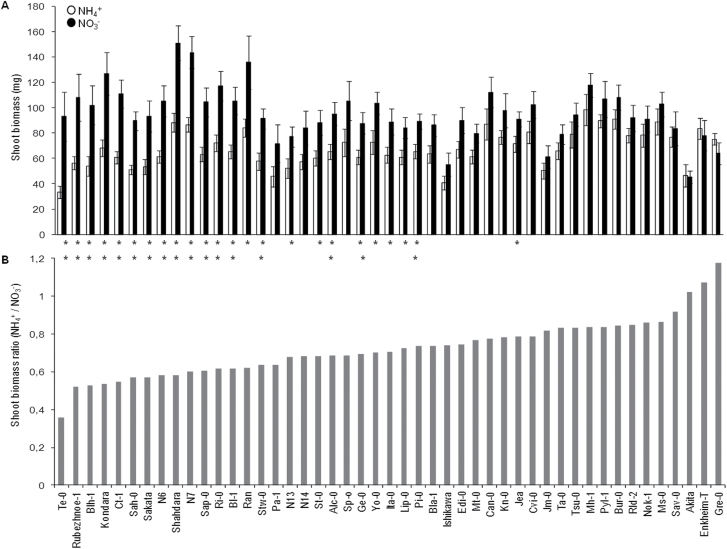
Natural variation of *Arabidopsis thaliana* growth under nitrate or ammonium as N source. (A) Shoot biomass. (B) Ratio between shoot biomass under NH_4_^+^ and NO_3_^–^ nutrition. Means and standard errors were calculated from 8–12 plants. Significant differences between shoot biomass under ammonium compared with nitrate nutrition are indicated for each accession (*P<0.05; **P>0.01).

To evaluate NUE with ammonium as the sole N source, *Arabidopsis* rosette biomass was compared after 3 weeks of growth under 1mM NH_4_^+^ [0.5mM (NH_4_)_2_SO_4_] or 1mM NO_3_^–^ [0.5mM Ca(NO_3_)_2_], and the ratio between shoot biomass under NH_4_^+^ and NO_3_^–^ conditions (SB NH_4_^+^/NO_3_^–^) was used to estimate ammonium tolerance as it has been previously used in other studies (Cruz *et al.*, 2006; Ariz *et al.*, 2011). In general, *Arabidopsis* is a species sensitive to NH_4_^+^ and nearly every ecotype analysed showed shoot biomass inhibition in response to NH_4_^+^. Twenty-four out of the forty-seven accessions analysed experienced a significant growth inhibition upon NH_4_^+^ nutrition (Fig. 1A). The accession Te-0 was the one showing the lowest SB NH_4_^+^/NO_3_^–^ ratio (<0.4), which was significantly lower than that of the next most sensitive accession to NH_4_^+^ (Rubenzhnoe-1; SB NH_4_^+^/NO_3_^–^ 0.56). Only three accessions had an SB NH_4_^+^/NO_3_^–^ ratio >1, but without significant differences between both types of nutrition (Akita, Enkheim-T, and Gre-0; Fig. 1B). Overall, intraspecific shoot growth variability under a contrasting N source is evident by the use of this collection of accessions (Fig. 1B).

**Fig. 2.**
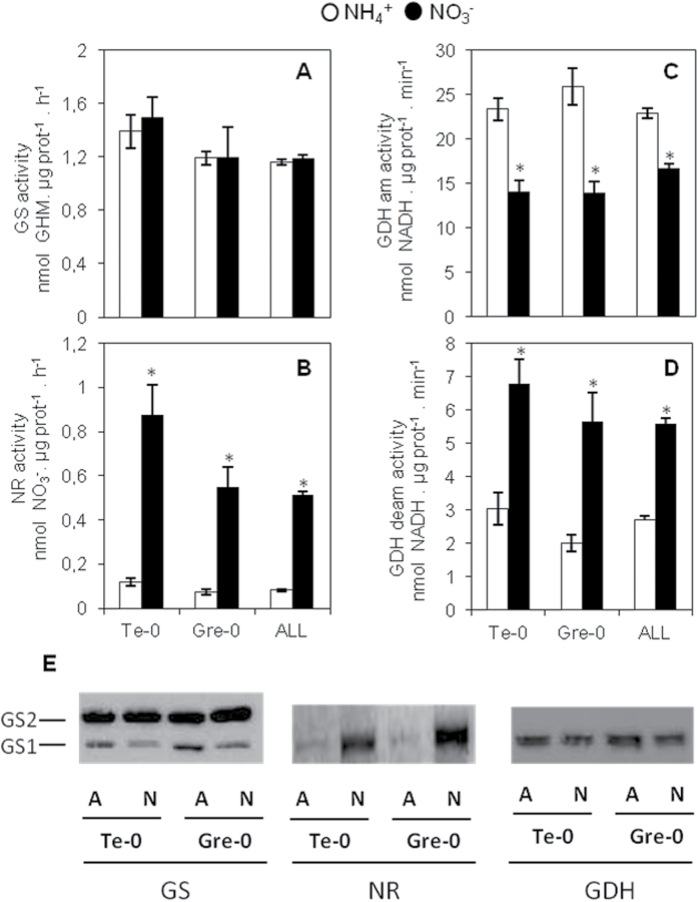
Enzyme activities of Te-0 and Gre-0 accessions and the mean of every accession (ALL) for (A) GS, (B) NR, (C) GDHam, and (D) GDH deam, and (E) western blot of GS, GDH, and NR for Te-0 and Gre-0 accessions grown under ammonium or nitrate nutrition. An asterisk indicates a significant difference for *P*<0.05 (*n*=6).

The content of ammonium and free amino acids (Supplementary Table S1 available at *JXB* online) as well as NR, GS, and GDH enzyme activities (Supplementary Table S1) were determined. GDH activity was measured both in the aminating (GDHam) and in the deaminating (GDHdeam) direction. Regarding NH_4_^+^ content, overall, plants under NH_4_^+^ nutrition contained significantly more NH_4_^+^ compared with plants fed with NO_3_^–^. Eight accessions (Enkheim-T, Gre-0, Ishikawa, Jea, Ms-0, Ran, Ta-0, and Tsu-0) did not show significant differences between both treatments (Supplementary Table S1). Amino acid content followed a similar trend to NH_4_^+^ content (Supplementary Fig. S1) and every accession under NH_4_^+^ nutrition contained significantly more amino acids compared with under NO_3_^–^ nutrition (Supplementary Table S1, Fig. S1). Concerning the enzyme activities, as expected, every accession under NO_3_^–^ nutrition had a higher NR activity (Fig. 2B; Supplementary Table S1). GS activity was similar for every accession under both forms of nutrition, except for Mt-0 and Ct-1 that showed a slightly higher GS activity under NO_3_^–^ nutrition and for Rld-2, N7, and N14 that experienced a small increase under NH_4_^+^ nutrition (Fig. 2A; Supplementary Table S1). GDHam activity was higher under NH_4_^+^ nutrition in 35 out of the 47 accessions. In contrast, GDHdeam activity was higher under NO_3_^–^ nutrition in every accession except for Akita, Ishikawa, Rld-2, Pa-1, and Sah-0, which did not show significant differences between both forms of nutrition (Fig. 2C, D; Supplementary Table S1).

**Fig. 3.**
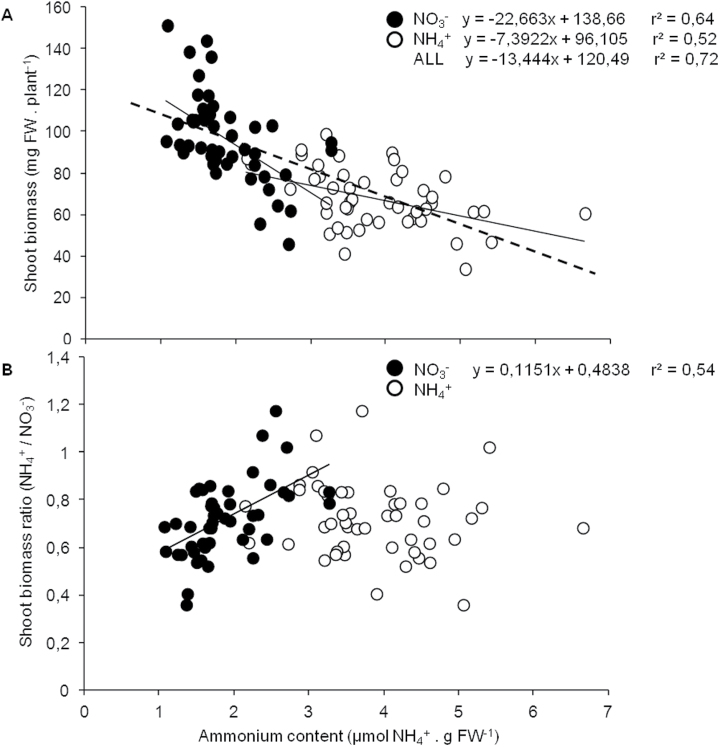
Scatter plots of ammonium content (horizontal axis) versus (A) shoot biomass and (B) the ratio between shoot biomass under NH_4_^+^ and NO_3_^–^. Linear regression and Pearson *r* ^2^ are given only if *P* was <0.05.

To investigate the connectivity between the different parameters, a Pearson correlation analysis was performed for each pair of parameters. Values are given for the correlation coefficient (*r* ^2^) and the significance (*P*). The results are presented separately for the plants grown under NH_4_^+^ (Table 1) and NO_3_^–^ nutrition (Table 2). Shoot biomass under both ammonium and nitrate nutrition was negatively correlated with NH_4_^+^ and free amino acid content (Tables 1, 2; Fig. 3A), which is reasonable because it could mean that part of the absorbed N is not being used for growth, and ammonium accumulation inside plant tissues is known to be deleterious for plant performance (Britto and Krontzuker, 2002; Ludewig et al., 2007). None of the parameters determined in NH_4_^+^-fed plants showed any correlation with the SB NH_4_^+^/NO_3_^–^ ratio (Table 1). In contrast, in NO_3_^–^-fed plants, NH_4_^+^ and amino acid content, together with GDHam activity, were positively correlated with the SB NH_4_^+^/NO_3_^–^ ratio (Table 2).

**Table 1.** Pearson correlations between the determined parameters in Arabidopsis thaliana plants under NH_4_^+^ nutritionSB indicates the shoot biomass, and SB NH_4_^+^/NO_3_^–^ denotes the shoot biomass ratio between NH_4_^+^- and NO_3_^–^-fed plants.

		SB NH_4_^+^/NO_3_^–^	SB	NH_4_^+^	Amino acids	NR activity	GS activity	GDHam activity	GDHdeam activity
SB NH_4_^+^/NO_3_^–^	*r* ^2^	1							
	*P*								
SB	*r* ^2^	0.427**	1						
	*P*	**0.002**							
NH_4_^+^	*r* ^*2*^	–0.144	–0.447**	1					
	*P*	0.328	0.001						
Amino acids	*r* ^2^	–0.001	–0.405**	0.554**	1				
	*P*	0.997	**0.004**	**0.000**					
NR activity	*r* ^2^	–0.014	–0.192	0.099	0.048	1			
	*P*	0.926	0.192	0.505	0.744				
GS activity	*r* ^2^	0.105	–0.016	–0.118	0.002	0.248	1		
	*P*	0.476	0.913	0.426	0.988	0.089			
GDHam activity	*r* ^2^	0.212	0.011	0.327*	0.321*	0.056	0.054	1	
	*P*	0.149	0.940	**0.023**	**0.026**	0.704	0.717		
GDHdeam activity	*r* ^2^	0.136	–0.052	0.265	0.305*	0.139	0.010	0.687**	1
	*P*	0.355	0.723	0.069	**0.035**	0.346	0.948	**0.000**	

**Table 2.** Pearson correlations between the determined parameters in *Arabidopsis thaliana* plants under NO_3_^–^ nutritionSB indicates the shoot biomass, and SB NH_4_^+^/NO_3_^–^ denotes the shoot biomass ratio between NH_4_^+^- and NO_3_^–^-fed plants.

		SB NH_4_^+^/NO_3_^–^	SB	NH_4_^+^	Amino acids	NR activity	GS activity	GDHam activity	GDHdeam activity
SB NH_4_^+^/NO_3_^–^	*r* ^2^	1							
	*P*								
SB	*r* ^2^	–0.524**	1						
	*P*	**0.000**							
NH_4_^+^ content	*r* ^2^	0.547**	–0.566**	1					
	*P*	**0.000**	**0.000**						
Amino acid content	*r* ^2^	0.478**	–0.544**	0.496**	1				
	*P*	**0.001**	**0.000**	**0.000**					
NR activity	*r* ^2^	0.124	–0.138	0.340*	0.085	1			
	*P*	0.403	0.349	**0.018**	0.567				
GS activity	*r* ^2^	0.029	–0.105	0.139	–0.014	0.335*	1		
	*P*	0.845	0.478	0.346	0.927	**0.020**			
GDHam activity	*r* ^2^	0.438**	–0.238	0.389*	0.326*	0.162	0.066	1	
	*P*	**0.002**	0.103	**0.006**	0.024	0.271	0.655		
GDHdeam activity	r^2^	0.048	0.146	0.078	–0.187	0.220	0.489**	0.156	1
	p	0.744	0.321	0.596	0.204	0.133	**0.000**	0.288	

Regarding the enzyme activities, in NH_4_^+^-fed plants, neither GS nor NR activity showed any correlation with any of the parameters determined (Table 1). GDHam and GDHdeam activities were positively correlated with each other, suggesting that when a genotype shows high GDH activity, it occurs in both the aminating and deaminating directions. Both GDHam and GDHdeam activities were positively correlated with amino acid content; however, only GDHam activity was positively correlated with NH_4_^+^ content (Table 1). In NO_3_^–^-fed plants, NR activity was positively correlated with NH_4_^+^ content and with GS activity (Table 2). In addition, GS activity was also correlated with GDHdeam activity. Interestingly, and similarly to NH_4_^+^-fed plants, in NO_3_^–^-fed plants GDHam activity was also correlated with ammonium and amino acid content (Table 2).

In order to better understand the relationships between the SB NH_4_^+^/NO_3_^–^ ratio and the different determined parameters, a multiple regression full model and AIC best model (AIC-selected) were applied. The full model only indicated a significant selection for the ammonium content in NO_3_^–^-fed plants (Table 3) and explained 23% of the variance in SB NH_4_^+^/NO_3_^–^. In the best model, the percentage of the variance in SB NH_4_^+^/NO_3_^–^ explained increased up to 38%. From the four traits retained in the best model (ammonium content in both NH_4_^+^- and NO_3_^–^-fed plants; amino acid content in NO_3_^–^-fed plants; and NR activity under NH_4_^+^ nutrition), NH_4_^+^ and amino acid accumulation in NO_3_^–^-fed plants were significantly retained. Interestingly, NH_4_^+^ content explained 53% of the best model.

**Table 3.** Full and Akaike’s information criterion (AIC)-selected best multiple regression models of *Arabidopsis thaliana* ammonium tolerance based on the ratio of the rosette biomass between plants grown under NH_4_^+^ or NO_3_^–^ nutritionSelection gradients (β) and standard errors (SE) are presented along with *P*-values.

Trait	Treatment	SB NH_4_^+^/NO_3_^–^			
		Full model		AIC-selected best model	
		β ±SE	*P*-value	β ±SE	*P*-value
NH_4_^+^	A	–0.037±0.029	0.214	–0.033±0.020	0.108
NH_4_^+^	N	**0.155±0.045**	**0.002**	**0.106±0.041**	**0.002**
NO_3_^–^	A	–0.001±0.003	0.848	–	–
NO_3_^–^	N	–0.001±0.002	0.651	–	–
Amino acids	A	0.001±0.002	0.630	–	–
Amino acids	N	0.010±0.005	0.081	**0.008±0.004**	**0.040**
NR activity	A	–0.882±1.064	0.412	–1.266±0.795	0.119
NR activity	N	–0.031±0.183	0.867	–	–
GS activity	A	–0.053±0.172	0.760	–	–
GS activity	N	–0.042±0.143	0.773	–	–
GDHam activity	A	–0.005±0.009	0.594	–	–
GDHam activity	N	0.010±0.008	0.215	–	–
GDHdeam activity	A	0.021±0.048	0.669	–	–
GDHdeam activity	N	–0.004±.0.022	0.852	–	–
		*r* ^2^ 0.23		*r* ^2^ 0.38	

Significant selection gradients are presented in bold.

A, ammonium-fed plants; N, nitrate-fed plants.

The same analysis was performed for the shoot biomass under both forms of nutrition. For NH_4_^+^-fed plants, the models only indicated selection for ammonium content, and both the full and best models only explained 19% of the variance in shoot biomass (Supplementary Table S3 at *JXB* online). For NO_3_^–^-fed plants, both the full and the best model explained 39% of the variance in shoot biomass. The full model indicated selection for ammonium and amino acid content (Supplementary Table S2), and both models significantly retained the ammonium and amino acid content (Supplementary Table 2).

According to the importance given by both Pearson correlations and the multiple regression models, the correlation of ammonium content both with shoot biomass and with SB NH_4_^+^/NO_3_^–^ was illustrated (Fig. 3). As shown by Pearson analysis (Tables 1, 2), ammonium content was negatively correlated with shoot biomass under both NH_4_^+^ and NO_3_^–^ nutrition (Fig. 3A). Interestingly, and as suggested by the multiple regression model, only the ammonium content in NO_3_^–^-fed plants was correlated with the SB NH_4_^+^/NO_3_^–^ ratio (Fig. 3B).

To understand further the behaviour of the N-assimilating enzymes determined, the enzyme activities were illustrated and western blotting analysis was performed for the accessions Te-0 and Gre-0, the most sensitive and tolerant accessions to ammonium, respectively (Fig. 3). This analysis did not show any difference for any of the three enzymes under both forms of nutrition. However, it was useful to ascertain that although there were no significant differences in GS activity, the GS1 isoform content was clearly accumulated upon ammonium nutrition (Fig. 2E). NR protein content, in agreement with NR activity, was dramatically induced in NO_3_^–^-fed Te-0 and Gre-0 plants. Finally, GDH content increased in NH_4_^+^-fed plants, according to the increase in GDHam activity (Fig. 2C). In contrast, although GDH was induced upon ammonium nutrition, as described above, GDHdeam activity increased in NO_3_^–^-fed plants (Fig. 2D). However, it must be noted that under NH_4_^+^ nutrition, the average GDHam activity was around eight times higher than the GDHdeam activity, whilst under NO_3_^–^ nutrition GDHam activity was about three times higher than GDHdeam activity.

## Discussion

Plant response to N availability depends on the genotype, the N source, and N fertilization level, and the limiting steps in N metabolism are different at low and high N supply (Chardon *et al.*, 2012; Xu *et al.*, 2012). Overall, NUE is higher when N supply is limiting. In general, adaptation to low N environments is challenging to most cultivars, because they have been selected under high-nutrient environments but plants in natural field conditions are faced with environmental changes where N availability varies and the better NUE under low N conditions is a competitive advantage (Kant *et al.*, 2011). Moreover, reducing N fertilizer input in the soil while maintaining productivity is an unavoidable strategy to reduce agricultural impact on the environment. Thus, and taking into account that *Arabidopsis* and the *Brassicaceae* family have been described as very susceptible to ammonium nutrition, in this work, a low N dose (1mM) was used. Because of this high sensitivity, most of the studies related to ammonium toxicity in *Arabidopsis* have been performed with mixed nutrition, and thus long-term ammonium-based nutrition studies involve the use of a low ammonium concentration.

Approaches based on intraspecific natural variation have become an important means to study plants adaptation. Regarding nitrate nutrition, studies based on natural variation have already been used in several species including maize (Coque and Gallais, 2007) and rice (Namai *et al.*, 2009). *Arabidopsis* natural variation has also been studied under limiting and ample nitrate supply (North *et al.*, 2009; Chardon *et al.*, 2010) and to evaluate the capacity of different genotypes for N remobilization during seed filling (Masclaux-Daubresse and Chardon, 2011). In contrast, studies focused in intraspecific variation of N use with ammonium as the sole N source are more scarce, although examples exist, studying, among others, four maize cultivars (Schortemeyer *et al.*, 1997), a collection of rice inbred lines (Obara *et al.*, 2010), and four pea cultivars (Cruz *et al.*, 2011). In this work, data from 47 natural accessions of *Arabidopsis* were collected and several traits related to N metabolism were measured to determine the natural variation of *Arabidopsis* growth and N metabolism (ammonium and amino acid content, and NR, GS, and GDH enzyme activities) under two different N sources (nitrate or ammonium). Biomass is considered as the best indicator of plant performance because it integrates every aspect of plant metabolism, from nutrient uptake to its assimilation, and the ratio of the shoot biomass under ammonium versus nitrate nutrition was considered here as an indicator of the plant’s tolerance/sensitivity to ammonium, as it has previously been used in other works (Cruz *et al.*, 2006; Ariz *et al.*, 2011). *Arabidopsis* accession N1438 grown under 2.5mM NH_4_^+^ for 21 d showed three times less biomass compared with plants grown under NO_3_^–^, and the authors suggested ionic imbalance as a major cause of this toxicity (Helali *et al.*, 2010). Similarly, Hoffman et al. (2007) reported a retardation of *Arabidopsis* Col-0 seedling growth under NH_4_^+^ nutrition compared with NO_3_^–^ nutrition. The present study confirms an overall sensitivity of *Arabidopsis* to ammonium, since, out of the 47 genotypes, 44 had a ratio <1 (23 accessions showing significant differences in shoot biomass between both forms of nutrition). However, this study highlights large intraspecific variation of ammonium tolerance expressed as SB NH_4_^+^/NO_3_^–^, which varied between 0.36 and 1.18. These values are in agreement with the values registered by Ariz *et al.* (2011) working with seven different species and ammonium concentrations. Thus, the present study, working with a low ammonium concentration, reveals a similar degree of intraspecific *Arabidopsis* ammonium tolerance variability to the interspecific degree of ammonium tolerance variability. This underscores the high variability within a single species and the power of natural variation approaches for plant adaptation studies.

### Ammonium accumulation affects plant growth

‘Excessive’ ammonium accumulation is toxic to cells. However, the concept of ‘excessive’ is extremely variable depending on the plant species and on the soil NH_4_^+^ concentration. In fact, ammonium toxicity is considered to be ‘universal’ even in species labelled as ‘NH_4_^+^ specialists’ (Li *et al.*, 2014). Excess ammonium causes an imbalance in, among others, pH homeostasis, ionic equilibrium, and primary metabolism (Britto and Kronzucker, 2002). Ammonium accumulation might derive from its direct uptake but also from amino acid deamination, protein degradation, and photorespiration. To prevent the cytosol from ammonium overload, plants deploy different strategies including AMT-type ammonium transporter regulation (Lanquar *et al.*, 2009) or increasing ammonium assimilation (Setien *et al.*, 2013). In the present work, as expected, NH_4_^+^-fed plants accumulated more NH_4_^+^ and amino acids than NO_3_^–^-fed plants and this NH_4_^+^ accumulation was negatively correlated with *Arabidopsis* rosette biomass (Fig. 3A). Interestingly, this correlation was found for plants grown under both forms of nutrition, suggesting that ammonium accumulation negatively influences plant growth even under nitric nutrition. NH_4_^+^ accumulation under low N supply might be due to a lack of proper carbohydrate supply for ammonium assimilation or to the toxicity caused by the excess NH_4_^+^ as stated above. To the authors’ knowledge, this is the first time that a correlation between plant shoot growth under NO_3_^–^ as sole N source and the accumulation of NH_4_^+^ in leaves has been reported, which provides evidence of the extreme sensitivity of *Arabidopsis* to ammonium.

Regarding the SB NH_4_^+^/NO_3_^–^ ratio, of the parameters determined, only ammonium, amino acid content, and GDHam activity from NO_3_^–^-fed plants showed a significant correlation (Table 2). Multiple regression full and best models retained ammonium and amino acid content, which both show a strong correlation (Supplementary Fig. S1 at *JXB* online), as significant factors explaining the variation in the SB NH_4_^+^/NO_3_^–^ ratio (Table 3). Interestingly, the NH_4_^+^ content of NH_4_^+^-fed plants did not show any significant correlation with the SB NH_4_^+^/NO_3_^–^ ratio. Thus, the fact that NO_3_^–^-fed plants with a higher NH_4_^+^ content present a smaller rosette biomass (Fig. 3A) could explain the relationship between ammonium content of NO_3_^–^-fed plants and the SB NH_4_^+^/NO_3_^–^ ratio (Fig. 3B). Alternatively, it can be speculated that evolutionarily a plant that under NO_3_^–^ nutrition is able to accumulate more ammonium could be genetically better adapted to an ammonium-based nutrition.

### Role of NR, GS, and GDH in *Arabidopsis* response to ammonium

NO_3_^–^ absorbed from the nutrient solution is reduced to ammonium, whereas in NH_4_^+^-fed plants this step is bypassed and ammonium is directly assimilated for plant growth. As expected, NR activity was induced upon NO_3_^–^ exposure but it was not related to differential plant growth. Indeed, NR or nitrite reductase overexpression in tobacco, potato, or *Arabidopsis* did not increase plant biomass, thus nitrate reduction does not seem to be a limiting step for plant growth (Pathak et al; 2008; Masclaux-Daubresse *et al.*, 2010). Ammonium assimilation in normal conditions in plants mainly occurs via the GS/GOGAT cycle. There are two different GS isoforms. GS1 is encoded by five genes in *Arabidopsis* and functions primarily in assimilating ammonia during nitrogen remobilization. GS2 is encoded by a single gene in *Arabidopsis* and has been involved in assimilating the ammonia coming from nitrate reduction or photorespiration (Xu *et al.*, 2012). In general, plants with higher GS activities are considered more tolerant to ammonium, and Cruz *et al.* (2006) showed a relationship between GS activity in the dark and ammonium tolerance. In this work, no difference in GS activity was found in almost every accession between NH_4_^+^- and NO_3_^–^-fed plants (Fig. 2A; Supplementary Table S2 at *JXB* online) and there was no correlation between GS activity and shoot biomass in plants under both forms of nutrition (Tables 1, 12). A western blot analysis was performed in two accessions with contrasting ammonium tolerance (Te-0 and Gre-0), and in both cases there was a clear accumulation of the GS1 isoform in response to ammonium nutrition. Overall, total GS activity does not seem to be crucial for ammonium tolerance in *Arabidopsis*; however, GS1 could have an important role when ammonium is supplied as the N source. Moreover, out of the five genes encoding GS1 in *Arabidopsis* GS1;2 is the most highly expressed in leaves and it is induced by ammonium (Lothier *et al.*, 2011). Indeed, an *Arabidopsis* mutant lacking GS1;2 expression exhibited reduced growth under a 7 d ammonium treatment compared with the wild type (Lothier *et al.*, 2011). Similarly, a rice mutant in the *GS1;1* gene was also more sensitive upon ammonium nutrition (Kusano *et al.*, 2011). Thus, it remains to be determined whether GS1;2 and the rest of the GS isozymes are related to *Arabidopsis* variability under ammonium nutrition. Also, very recently root NADH-GOGAT has been suggested to play an important role in ammonium assimilation under ammonium nutrition (Konishi *et al.*, 2014s).

GDH is able to catalyse the *in vitro* reversible amination of 2-oxoglutarate to glutamate. *In vivo*, the existence of the N assimilating capacity of GDH is controversial and in the last years evidence has been accumulating in favour of the major role of GDH deamination, for example by the use of ^15^N-nuclear magnetic resonance (NMR) labelling studies showing that there was no direct incorporation of ammonia into glutamate when GS was inhibited (Labboun *et al.*, 2009; Tercé-Laforgue et al., 2013). However, although in unstressed plants GDH ammonia assimilating capacity seems to be negligible, it appears that under stress conditions and under ammonium nutrition, GDH could incorporate NH_4_^+^ (Skopelitis; 2006; Setien *et al.*, 2013). In the present study, a contrasting behaviour of GDH activity was found. GDHam activity was generally induced upon NH_4_^+^ exposure whereas GDHdeam activity was repressed (Fig. 2C, D; Supplementary Table S1 at *JXB* online). Moreover, in both NH_4_^+^- and NO_3_^–^-fed plants ammonium and amino acid contents were positively correlated with GDHam activity, and not with GDHdeam activity (Tables 1, 12). Thus, the present data suggest that NH_4_^+^ accumulation might be stimulating the ammonium-incorporating capacity of GDH rather than being a consequence of NH_4_^+^ release associated with GDH glutamate deamination. Nevertheless, experiments designed to ascertain the actual GDHam activity in conditions of plant growth under an exclusive ammoniacal nutrition, such as by ^15^N-NMR labelling, are necessary.

GDH is traditionally accepted to form seven isoenzymes composed of α and β homo- or heterodimers. Recently, the existence in *Arabidopsis* of a third gene encoding a γ subunit has been shown (Fontaine *et al.*, 2012). However, the activity of this γ isoenzyme was exclusively from root (Fontaine *et al.*, 2012), which is in line with the hypothesis that each of the GDH subunits may have specific biological functions (Purnell *et al.*, 2005; Tercé-Laforgue et al., 2013). In the present work, after SDS–PAGE, GDH was accumulated under ammonium nutrition (Fig. 2E). An accumulation of GDH polypeptides has already been reported in several species including wheat (Setien *et al.* 2013), pea (Ariz *et al.*, 2013), and tomato (Setien *et al.* 2014). The overall data indicate a key role for GDH in *Arabidopsis* under NH_4_^+^ nutrition.

### Concluding remarks and future prospects

Overall, the results obtained in this work reveal that there exists high natural variation in *A. thaliana* growth as a function of the N source. This variation was partially due to the differential tissue NH_4_^+^ and amino acid accumulation in both NO_3_^–^-fed and NH_4_^+^-fed plants. Similarly, significant natural variability was detected in NH_4_^+^ tolerance expressed as the SB NH_4_^+^/NO_3_^–^ ratio, and, interestingly, NH_4_^+^ accumulation in NO_3_-fed plants was the parameter showing the highest relevance, which may indicate an evolutionary adaptation suggesting that plants that under NO_3_^–^ nutrition are able to accumulate more ammonium could be genetically better adapted to an ammonium-based nutrition. Although plant NH_4_^+^ assimilation capacity is known to be a key aspect for ammonium tolerance, GS and GDH activity does not seem to be responsible for the variability shown in *A. thaliana*. However, the modulation of GDH activity as a function of the supplied N source was clearly observed, which suggests an important role for this enzyme in NH_4_^+^ assimilation. Similarly, the observed higher content of the GS1 isoform in NH_4_^+^-fed plants could also contribute to NH_4_^+^ assimilation. The quality of the root system has also been suggested partly to explain the differences in nitrogen uptake and NUE (Loudet *et al.*, 2005). Furthermore, several works have highlighted the importance of the root in NH_4_^+^ tolerance (Setien et al., 2013, 2014, Kojima *et al.*, 2014). Thus, future works dealing with root metabolism will be useful to ascertain whether N assimilation in this organ could be related to the natural variability in NH_4_^+^ tolerance in *A. thaliana*. Also, approaches using larger *A. thaliana* natural populations in combination with genome-wide association studies (Atwell *et al.*, 2010) will no doubt be very helpful in elucidating the genetic basis underlying the *Arabidopsis* intraspecific variability in ammonium tolerance.

## Supplementary data

Supplementary data are available at *JXB* online.

Figure S1. Scatter plots of amino acids versus ammonium content of leaves of *Arabidopsis thaliana* grown under NH_4_^+^ and NO_3_^–^.

Table S1. Ammonium and amino acid content and enzyme activities: whole data set.

Table S2. Full and Akaike’s information criterion (AIC)-selected best multiple regression models of *Arabidopsis thaliana* rosette biomass grown under NH_4_^+^ or NO_3_^–^ nutrition.

Supplementary Data

## Abbreviations:

GDH: glutamate dehydrogenase
GDHam: glutamate dehydrogenase aminating
GDHdeam: glutamate dehydrogenase deaminating
GS: glutamine synthetase
NR: nitrate reductase
NUE: nitrogen use efficiency
SB: shoot biomass.
