# Supplementary material for: Exploring ammonium tolerance in a large panel of Arabidopsis thaliana natural accessions
Source: J Exp Bot. 2014 Sep 9;65(20):6023–33. doi: 10.1093/jxb/eru342 (PMC4203136; doi:10.1093/jxb/eru342)
Supplement: Supplementary Data [file supp_65_20_6023__index.html]

Exploring ammonium tolerance in a large panel of Arabidopsis thaliana natural accessions — Exploring ammonium tolerance in a large panel of Arabidopsis thaliana natural accessions — Exploring ammonium tolerance in a large panel of Arabidopsis thaliana natural accessions — Supplementary Data 

# Exploring ammonium tolerance in a large panel of *Arabidopsis thaliana* natural accessions

## Supplementary Data

Data files

**Files in this Data Supplement:**

- Supplementary Data - Supplementary Data
- Supplementary Data - Supplementary Data
