# Supplementary material for: Exploring ammonium tolerance in a large panel of Arabidopsis thaliana natural accessions
Source: J Exp Bot. 2014 Sep 9;65(20):6023–33. doi: 10.1093/jxb/eru342 (PMC4203136; doi:10.1093/jxb/eru342)

Exploring ammonium tolerance in a large panel of *Arabidopsis thaliana* natural accessions

Asier Sarasketa, María Begoña González-Moro, Carmen González-Murua and Daniel Marino

Supplementary data

Tables S1, S2 – see separate file

Table S3. Full and Akaikes information criterion (AIC)-selected best multiple regression models of *Arabidopsis thaliana* rosette biomass under NH_4^+ or NO_3^- nutritions. Selection gradients (β) and standard errors (SE) are presented along with P-values. Significant selection gradients are presented in bold.

	**Biomass (NH_4^+-fed)**				**Biomass (NO_3^--fed)**			
	Full model		AIC-selected best model		Full model		AIC-selected best model	
	β ± SE	P value	β ± SE	P value	β ±SE	P value	β ± SE	P value
**NH_4^+**	**-6,011± 2,717**	**0,033**	**-7,382 ± 2,185**	**0,002**	**-12,27 ± 5,64**	**0,006**	**-14,307 ± 5,161**	**0,008**
**Amino acids**	-0,284 ± 0,183	0,128	-	-	**-1,246 ± 0,561**	**0,032**	**-1,474 ± 0,528**	**0,008**
**NR activity**	-94,95 ± 86,21	0,337	-	-	13,24 ± 20,19	0,316	-	-
**GS activity**	-3,081 ± 15,16	0,586	-	-	-22,84 ± 15,88	0,158	-	-
**GDHam activity**	0,792 ± 0,713	0,274	-	-	0,094 ± 0,757	0,902	-	-
**GDHdeam activity**	0,013 ± 4,160	0,824	-	-	4,523 ± 2,569	0,086	-	-
	r^2 0.19		r^2 0.19		r^2 0.39		r^2 0.39	

Figure S1. Scatter plots of amino acids versus ammonium content of leaves of *Arabidopsis thaliana* grown under NH_4^+ and NO_3^-. Linear regression equations and person r^2 are given.

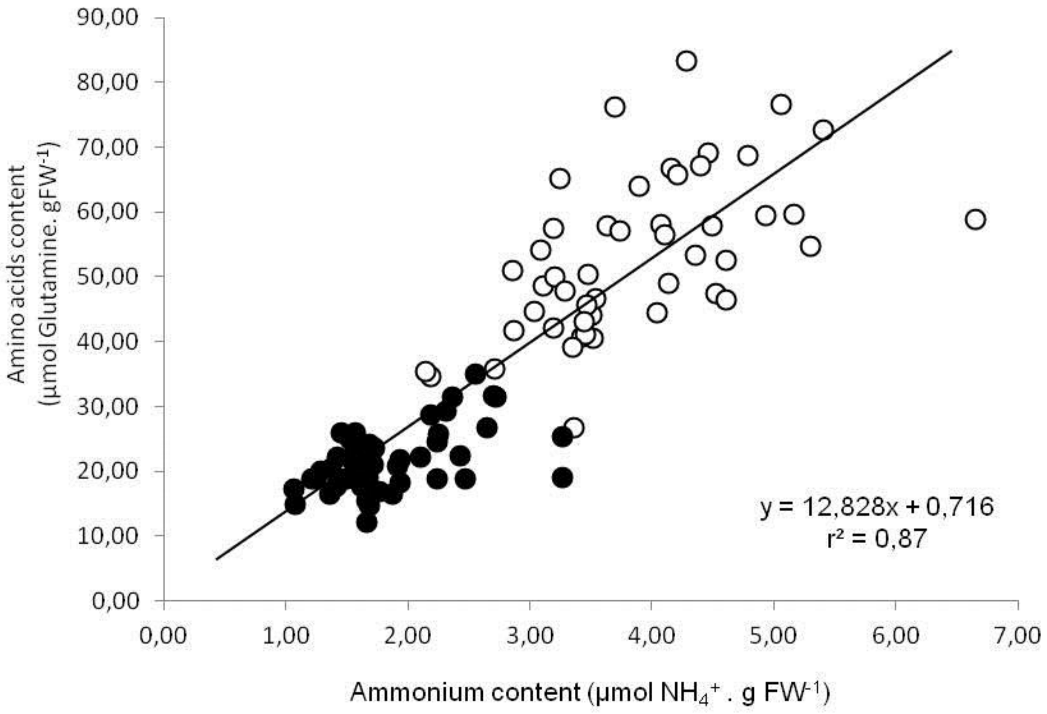

Supplement: Supplementary Data [file supp_eru342_jexbot127829_file002.pdf]
